# Prevention of acute kidney injury by intravenous sodium bicarbonate: the end of a saga

**DOI:** 10.1186/s13054-014-0672-0

**Published:** 2014-11-27

**Authors:** Helmut Schiffl

**Affiliations:** SRH Applied University of Health Sciences, Neue Str. 28-30, D-07548 Gera, Germany

The systematic review and meta-analysis of prospective randomized trials reported by Tie and colleagues [1] in a previous issue of *Critical Care* provide evidence that intravenous sodium bicarbonate (SBIC) administration does not reduce the incidence of cardiac surgery-associated (CSA) acute kidney injury (AKI) but prolongs the duration of mechanical ventilation and of hospital stay.

The conclusions of the authors are corroborated by a recent comprehensive systematic review [2] demonstrating that the administration of SBIC to patients at risk for CSA-AKI, contrast-induced nephropathy, septic AKI, or pigment nephropathy has no additional benefit compared with saline but adds to in-hospital morbidity and mortality.

The authors do not discuss the mechanisms underlying possible harms of SBIC. In cardiac patients, this type of fluid may precipitate volume overload and acute pulmonary edema. Alkalosis-induced hypoventilation may be associated with myocardial ischemia aggravating decreased cardiac contractility and may cause arrhythmia by inducing hypokalemia [3,4].

The principal intervention with proven efficacy for the prevention of AKI is adequate fluid administration. Definitively, SBIC is not the optimal fluid.

**Authors’ response**

Hong-Tao Tie, Qing-Chen Wu and Jing-Yuan Wan

We appreciate Schiffl’s insightful commentary, which encourages us to expand on the mechanisms of SBIC in the prevention of CSA-AKI.

The inefficacy of SBIC was due to the hypothesis that the significant increased power of hydrogen (PH) might not be adequate to prevent CSA-AKI. Nevertheless, the increased PH and subsequent disruption of homeostasis could cause possible harms. Firstly, arrhythmia and hypoventilation via inducing hypokalemia and alkalosis impair the cardiac function, as indicated by Schiffl. Moreover, hypocapnia by inducing alkalosis could reduce the cerebral blood flow and result in seizures and even coma or death [5]. Secondly, SBIC administration could impair the oxygenation and subsequently exacerbate the ischemia-reperfusion injury by aggravating the ischemia [4]. Thirdly, the induced intracellular alkalinization is associated with increased cell death, cell apoptosis, superoxide formation, pro-inflammatory cytokine release, blood lactate, and ketone bodies [6]. Finally, SBIC could cause decreased arterial blood pressure [7], an emergency situation for patients after cardiac surgery. Therefore, the risks overwhelm the benefits of SBIC for CSA-AKI prevention in patients undergoing cardiac surgery.

However, the hypothesis that SBIC administration may precipitate volume overload and acute pulmonary edema seems irrational because each patient received the same solution (for example, 5% dextrose) and the same amount of fluid volume in each study. In short, the possible harms of SBIC should not be attributed to volume overload and acute pulmonary edema.

## Abbreviations

AKI: acute kidney injury
CSA: cardiac surgery-associated
PH: power of hydrogen
SBIC: sodium bicarbonate
